# Bilateral TMJ Involvement in Rheumatoid Arthritis

**DOI:** 10.1155/2014/262430

**Published:** 2014-04-02

**Authors:** Pritesh B. Ruparelia, Deep S. Shah, Kosha Ruparelia, Shreyansh P. Sutaria, Deep Pathak

**Affiliations:** ^1^Department of Oral Medicine and Radiology, College of Dental Science and Research Centre, 47 Jai Ambika Society, Isanpur Road, Maninagar, Ahmedabad, Gujarat 380008, India; ^2^Department of Oral Medicine and Radiology, AMC Dental College, Khokhra, Ahmedabad, Gujarat, India

## Abstract

Rheumatoid arthritis (RA) is a systemic inflammatory, slowly progressive disease that results in cartilage and bone destruction. Temporomandibular joint (TMJ) involvement is not uncommon in RA, and it is present in about more than 50% of patients; however, TMJ is usually among the last joints to be involved and is associated with many varied clinical signs and symptoms. Hence, RA of TMJ presents to the dentist with great diagnostic challenges. This report presents a case of RA with bilateral TMJ involvement with its classical radiographic findings and review literature.

## 1. Introduction

“Rheumatoid arthritis (RA) is a chronic inflammatory disease characterized by joint swelling, joint tenderness, and destruction of synovial joints, leading to severe disability and premature mortality [1, 2].”

The first recognized description of RA was made in 1800 by Dr. Augustin Jacob Landré-Beauvais of Paris [3]. A B Garrod in 1858 named the disease rheumatoid arthritis replacing the old terms arthritis deformans and rheumatic gout [3]. He is thus credited to make a distinction between rheumatoid arthritis, osteoarthritis, and gout [4]. In 1932 the International Committee on Rheumatism was formed which later became American Rheumatism Association and then American College of Rheumatology [4].

TMJ complaints are present in about more than 50% of patients of RA [4, 5]. TMJ is usually among the last joint to be involved and is associated with many clinical signs and symptoms of which pain is a major problem later leading to inflammation, limited movements, swelling (joint stiffness), and muscle spasm [6]. If it occurs in early age it may result in mandibular growth disturbance, facial deformity, and ankylosis and in adult these can vary from mild joint stiffness to total joint disruption with occlusal-facial deformity [7, 8].

The diagnosis of TMJ involvement in RA is exclusionary based on history, physical findings, radiographic study, and lab testing. Hence a multidisciplinary approach is necessary [8, 9].

The present paper reports a case of RA with bilateral TMJ involvement with its classical radiographic findings.

## 2. Case Report

**Figure 1:**
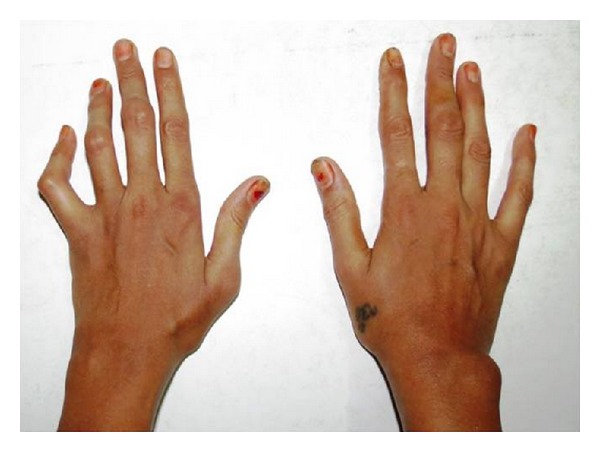
Showing minor joint deformity with stiffness of the interphalangeal joints of the hand.

A 29-year-old female patient complained of pain in front of ear bilaterally and discomfort during mouth opening since last 2 months. Associated complains reported anorexia, nervousness, fatigue, and weakness. Four weeks later she began to feel continuous throbbing pain in the joints which aggravated during chewing. Gradually the pain became very intense, making it difficult for the patient to open the mouth, associated with clicking sound while mouth opening, on the right side in front of ear. Other medical and surgical history revealed mild pain and stiffness of the joints of the hands and feet (Figure 1).

**Figure 2:**
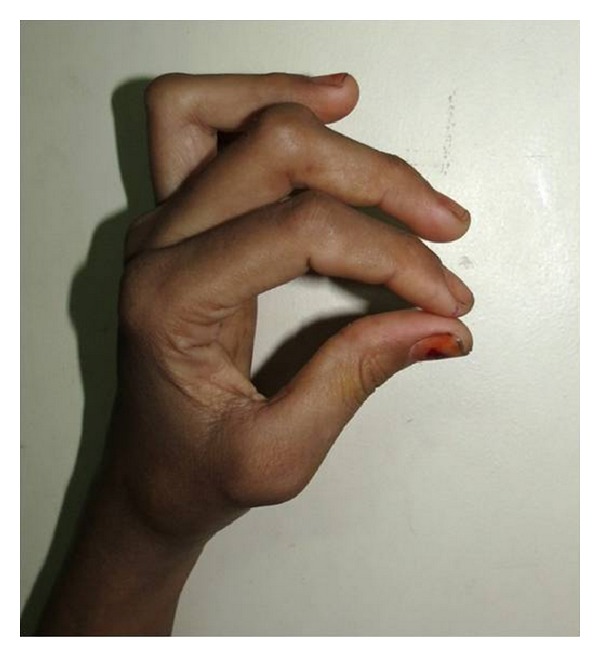
Showing swan neck deformity of left hand.

**Figure 3:**
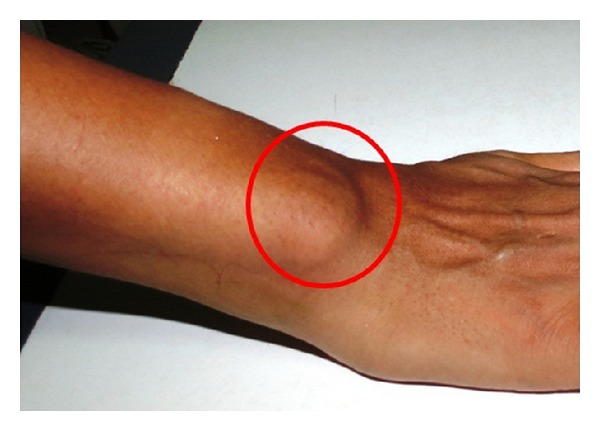
Showing presence of nodules at the right wrist joint on lateral aspect.

**Figure 4:**
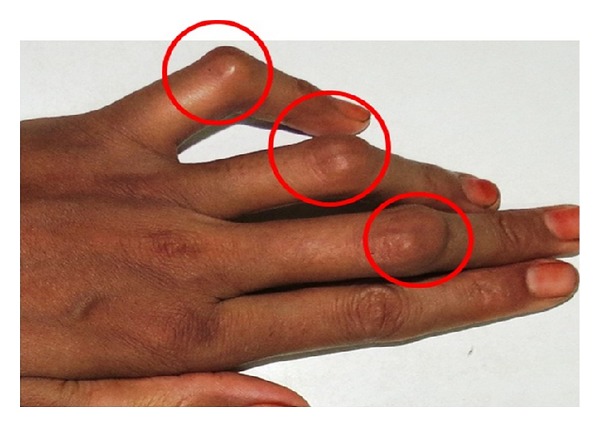
Showing presence of nodules on the interphalangeal joint at the middle, third, and fourth finger of left hand.

General examination revealed minor joints deformity and stiffness of the interphalangeal joints of the hand and feet, causing swan neck deformity of the fingers, which is a disabling deformity of wrist and fingers (Figure 2). Swelling was present on the interphalangeal joint at the middle, third, and fourth finger of left hand and on lateral aspect of the right wrist joint (Figures 3 and 4).

**Figure 5:**
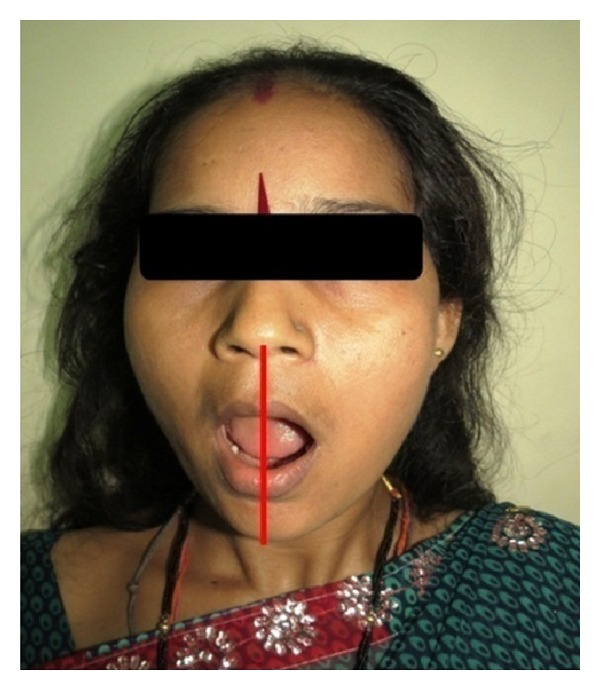
Showing deviation of mandible towards right side.

TMJ examination showed decreased movement and dull preauricular pain during function. Right preauricular depression with deviation of mandible to the same side (right) during opening. On palpation bilateral TMJ tenderness was present which was more pronounced on right side. Crepitus was elicited on right and left side of TMJ, more intense on right side, during mouth opening (Figure 5).

Based on the detailed history and clinical observations provisional diagnosis of bilateral TMJ involvement by RA was given with differential diagnosis of Gout, Osteoarthritis, Felty's syndrome, Still's Disease, Systemic Lupus Erythematosus (SLE), and Sjögren's syndrome. Then patient was subjected to radiologic and laboratory investigations.

**Figure 6:**
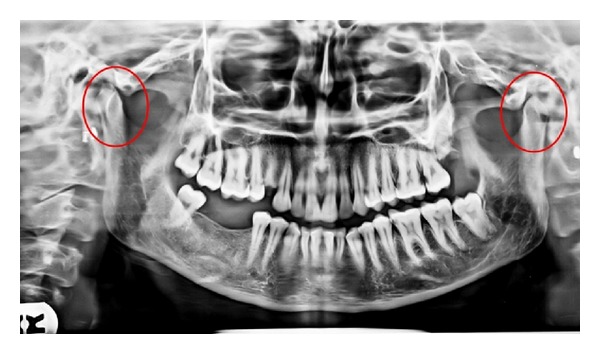
Panoramic view showing irregular erosion on right and left side of condyle with flattening of articular eminence.

**Figure 7:**
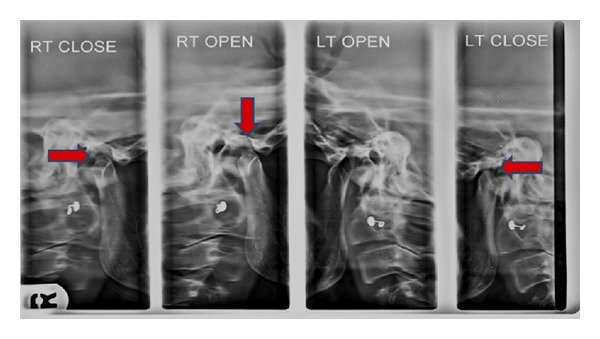
TMJ view showing erosive changes with lack of cortication of condyle and glenoid fossa with lack of translation of condyle.

**Figure 8:**
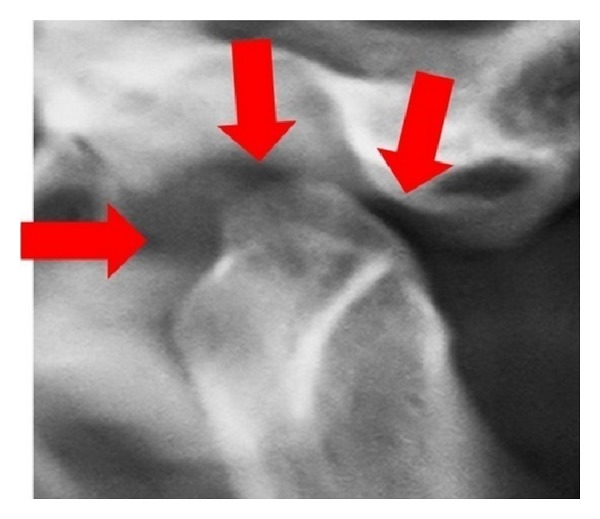
Showing presence of scooped out area of erosion in posterosuperior aspect of head of condyle giving appearance of mouthpiece of flute.

Panoramic view showed Irregular erosion on right and left side of condylar head with flattening of articular eminence (Figure 6). Digital view of TMJ OPG showed erosive changes with lack of cortication of posterosuperior surface of the right and left condyle and glenoid fossa. In open mouth position on right side head of the condyle appear beneath the articular eminence suggestive lack of translation of condyle (Figure 7). Presence of scooped out area of erosion in posterosuperior aspect of head of condyle giving appearance of “mouth piece of flute”. (Figure 8), a remarkable radiographic sign for RA.

**Figure 9:**
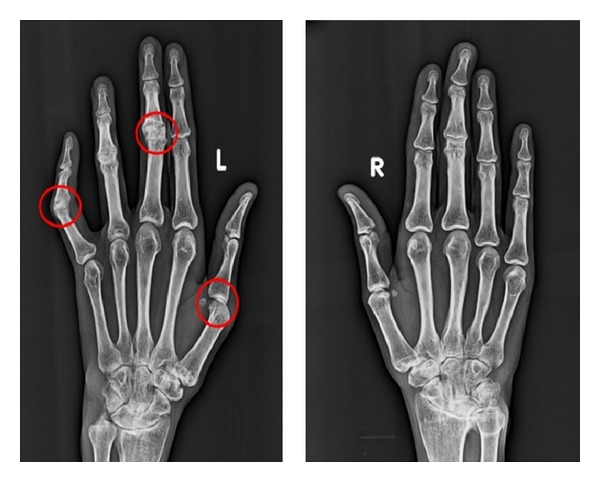
Hand wrist radiograph showing periarticular osteoporosis of interphalangeal joints of fingers.

Hand wrist radiographs revealed periarticular osteoporosis of interphalangeal joints of fingers and narrowing of joint space was observed in joints of hands (Figure 9).

Laboratory investigations revealed haemoglobin level of 9.2 mg/dL and raised ESR to 65 mm at the end of one hour, and leukocyte count 9,200/cmm of blood with normal differential count. RA Test for rheumatoid factor (RF) with latex agglutination method showed raised levels 73.40 IU/L (Ref value: up to 10 IU/L), and antinuclear antibody (ANA) test by indirect immunofluorescence method showed raised antinuclear antibody for RA. Serum uric acid level was normal, 4.9 mg% (Ref value is 2.4–5.7% for female).

Considering the above reports diagnosis of bilateral TMJ involvement by RA was confirmed. Subsequently, the patient was treated with NSAIDS and corticosteroids and later she was given oral and written instructions for heat and cold therapy and range of motion exercise several times with rest. Patient was recalled on follow-up visit after 2 weeks and reported relief of symptoms. She was further referred to rheumatologist for expert opinion and need for complete management of RA.

## 3. Discussion

Rheumatoid arthritis (RA) is a disease characterized by inflammation of the synovial membrane. Franks in 1969 reported that women are approximately three times more likely to be affected than men with RA. Abhijeet and Shirish in 2010 also concluded the same findings [11]. We also reported a case of RA in female patient.

Gynther and Tronje in 1998 reportedthat 80% of people with RA develop signs and symptoms of the disease at between 35 and 45 years of age; other studies by Voog et al. in 2003 and Ardic et al. in 2006 also reported the mean age within this range but we have reported a patient at early age of 30 years [12–14].

Kori and Stephen in 2012 reported that the clinical course may vary from mild joint discomfort of short duration to chronic polyarthritis, pain, and gross deformity of joints with swelling [3]. Chronic inflammation can lead to a loss of cartilage, erosion, and weakness of the bones and muscles, resulting in joint deformity, destruction, and loss of function, which were positive in the present case with added swan neck deformity of the fingers [15].

Franks reported that the common features in patients with rheumatoid arthritis were joint tenderness (70%) followed by joint crepitus (65%) and pain on mandibular function (60%) and decrease in mouth opening which all were positive in our case. The most characteristic clinical signs of rheumatoid arthritis are palpatory tenderness of the joint and crepitus that was also present in our case [16]. Systemically the disease may affect skin, blood vessels, eyes, pleura, lungs, peripheral nerves, and endocrine glands but there was no systemic involvement in the present case, as it may be diagnosed early [14].

Helenius et al. in 2005 reported that in rheumatoid arthritis, multiple joints of the body are commonly affected, TMJ being the last joint to be involved [15]. In the study by Abhijeet and Shirish in 2010 done in patients with rheumatoid arthritis, the mean duration of general disease was found to be 11.2 years while the duration of TMJ symptoms was found to be 1.7 years. These findings are similar to findings of Voog et al. [11, 13]. In the present case, though TMJ was involved after involvement of hand wrist joints, there were no other joints or systemic involvement, so it can be inferred that our case has been reported in relatively early phase of disease than is reported by many other authors.

*The American College of Rheumatology (ACR) 1987* laid down criteria for diagnosis of RA. However, these criteria are limited by poor sensitivity and specificity for classification of patients with early inflammatory arthritis as having rheumatoid arthritis [17]. They fail to identify individuals with very early arthritis who subsequently develop rheumatoid arthritis.

As a result of these concerns and developments, the* ACR and European League Against Rheumatism (EULAR)* have devised new classification criteria for early arthritis, which assess joint involvement, autoantibody status, and acute-phase response and symptom duration [10]. In 2010, they had given classification criteria for rheumatoid arthritis. In that minimum score of 6 out of 10 is required to put the diagnosis of definite RA [10]. In our case, the score was 7 (Table 1).

Ardic et al. in 2006 reported that the radiological changes of TMJ include cortical erosion, decreased joint space, deossification, sharpen pencil head or spiked deformity of the condylar head or mouth piece of flute deformity of condylar head, and subcortical cysts, which were all positive in our case except for subcortical cysts [2, 12].

Abhijeet and Shirish in 2010 reported that in patients with rheumatoid arthritis, the predominant finding was erosion of condyle (85%) followed by condylar sclerosis similar to study by Gynther and Tronje, Goupille et al., and Voog et al. [13, 14, 18]. Sclerosis is a sign of healing of joint in contrast to erosion, which indicated active bone disease. These findings are consistent with our findings [18].

Arnett et al. in 1988 stated that evidence of distinct erosion in panoramic tomogram was significantly associated with evidence of restricted condylar movement in a lateral panoramic radiograph [17]. This was also reported in the present case.

Goupille et al. in 1992 reported that erosive lesions may indicate acute or early changes whereas flattening and osteophyte formation may indicate late changes in TMJ [18]. As there is erosion in our case, it is suggestive of acute/early changes in TMJ.

Kurita et al. in 2004 reported that functional and parafunctional loading elicit adaptive and degenerative changes in load bearing joints including TMJ. In TMJ the anterosuperior part of the mandibular condyle and the posterior slope and inferior part of the articular eminence are assumed to bear the greatest load [19].Abhijeet and Shirish in 2010 also reported that erosion of superior part of mandibular condyle is most commonly seen in rheumatoid arthritis [11]. Present case reported posterosuperior portion of condyle to be affected.

Franks in 1969 stated that changes appear to occur in the anterior margin of condyle progressively and the destruction causes the condyle to resemble the sharpened pencil deformity [16]. Uotila in 1964 suggested that erosion only on anterior aspect resembles “the mouthpiece of the flute” [20]. But in our case erosion was on the posterosuperior aspect giving appearance of the mouthpiece of flute (may be termed as “reverse mouthpiece of flute appearance”).

## 4. Conclusion

There are many areas of interest to the dentist in treating TMJ involved with RA. However, signs and symptoms involving TMJ with RA should be suspected always. TMJ is usually among the last joints to be involved but functional examination of the TMJ may often reveal the first clinical symptoms and thus a dentist can help such patients for early diagnosis and management of their underlying polyarticular and multiorgan disorder not limited to orofacial area.

## Figures and Tables

**Table 1:** Showing scoring of our patient based on the 2010 American College Of Rheumatology classification criteria of rheumatoid arthritis [10].

			Patient score
(A)	Joint involvement		
	1 large joint	0	
	2–10 large joints	1	
	1–3 small joints (with or without involvement of large joints)	2	
	4–10 small joints (with or without involvement of large joints)	3	3
	>10 joints	5	
(B)	Serology (at least 1 test result is needed for classification)		
	Negative RF and negative ACPA	0	
	Low positive RF or low positive ACPA	2	2
	High positive RF or high positive ACPA	3	
(C)	Acute phase reactants (at least 1 test result is needed for classification)		
	Normal CRP and normal ESR	0	
	Abnormal CRP or abnormal ESR	1	1
(D)	Duration of symptoms		
	<6 weeks	0	
	>6 weeks	1	1
